# Proceedings and Debates of the Third National Quarantine and Sanitary Convention, Held April, 1859

**Published:** 1860-05

**Authors:** 


ART. IV.—*Proceedings and Debates of the Third National Quarantine and Sanitary Convention, held in the City of New York, April*, 1859. 8vo., pp. 728. New York, 1859.

THE Quaratine and Sanitary Convention, held at New York in April last, was in session four days, and if the ponderous volume before us is to be taken as any evidence, we may justly conclude that the meeting was one of great interest. That much earnest discussion was elicited is certain. The speeches of the learned Francis and of the no less learned La Roche are extremely elaborate, and evince no little skill at an attempt to show which is the better side of a grave and important question. The debate occasionally waxed quite hot, and yet were the blows of the adversaries dealt so gently and so gracefully that, as the weapons of their rhetoric descended, they fell harmlessly upon the head at which they were leveled. Fortunately, neither of the combatants could appropriate to himself the saying, "*Quid nisi victis labor.*"

The Report of the Committee on Quarantine, drawn up by Drs. Wilson Jewell, Francis Condie, and W. J. Wragg, embraces upwards of eighty pages, and exhibits an unusual amount of erudition and care in its preparation. The historical portion of the document, from the first of these able physicians, is a most painstaking production, distinguished for the extent and minuteness of its research, and is destined to occupy a permanent place in our quarantine and sanitary literature.

The Report on the Importance and Economy of Sanitary Measures was furnished by Dr. John Bell, of this city, and is the most elaborate article in the transactions, occupying not less than 199 pages. To say that the various topics comprehended in its scope have been ably discussed would not be doing the learned and indefatigable author justice. He has treated all with consummate ability, and his report will always be referred to as one of standard authority. There is no man in the country to whom the subject could have been more appropriately referred for patient and thorough elaboration. Whatever Dr. Bell touches with his pen he adorns.

In addition to the above reports, there is another, also of much merit, by the committee on "The Internal Hygiene of Cities," by Drs. Thomas Miller, E. M Snow, W. C. Van Bibber, R. D. Arnold, John H. Griscom, Henry G. Clark, and John Bell. Perhaps the most valuable parts of this document are those which refer to the labors of Morfitt, Muspratt, and Tardieu on the nature and use of disenfectants.

The President of the Convention was John H. Griscom, M.D., of New York, assisted by twelve vice-presidents, seven of whom were physicians, the others being lay delegates. Five secretaries officiated on the occasion. The list of delegates was very large and respectable, representing ten States and the District of Columbia. The deliberations of this distinguished body closed with a banquet at the Metropolitan Hotel, at which, judging from the "Proceedings," (and how is one who was not present to judge in any other way?) there must have been much hilarity with a great deal of anti-sanitary matter. Among the toasts that were drank, "woman," of course, was not forgotten, for there was probably no one present who did not recollect that he had, at one time or another, been *quarantined* by her.

A seraph all holy, she gladdens our way,
And garlands December with roses of May!
And shines the heart's star, wherever we roam,
The beacon of love, the angel of home.

The response to the above sentiment was very appropriately confided to Dr. Snow. He spoke with great *fervency*, and came very near *melting* away under his exertions.

We cannot but think that these conventions are destined to exert a salutary influence upon the health and happiness of the American people.

Their effects may be slow, but ultimately not the less certain. Public opinion has been fairly aroused, and the inevitable result will be further inquiry, the blessings of which no one can now predict.

We were particularly struck with the subjoined remarks in the address of Dr. Griscom, on taking the chair as the President of the Convention. We wish that the facts embraced in them could be written in letters of iron upon the forehead of every city in North America.

"The subject of quarantine has occupied the attention of mankind from time immemorial; but that of internal defenses against individual diseases, scarcely, until within a very few years. Yet what do we find to be the results in reference to these different circumstances? For example, in the City of New York, we have lost by *Cholera*, in twenty-five years, 12,300 inhabitants; by *Cholera Infantum*, 15,000; within twenty years, we have lost by *Hydrocephalus*, 12,400; by *Cholera Morbus*, 1400; by *Erysipelas*, 2100; by *Convulsions*, 20,000—*i.e.* convulsions belonging to and affecting infantile life. These are diseases which are generated chiefly within the city, and within the domicile itself, against which the city is defenseless; while from *Yellow Fever*, which can be kept out, and which is not generated among us, we have lost in *fifty years* only 600."

